# Social behavior in farm animals: Applying fundamental theory to improve animal welfare

**DOI:** 10.3389/fvets.2022.932217

**Published:** 2022-08-12

**Authors:** Victoria E. Lee, Gareth Arnott, Simon P. Turner

**Affiliations:** ^1^Animal Behaviour and Welfare, Animal and Veterinary Sciences Department, Scotland's Rural College (SRUC), Edinburgh, United Kingdom; ^2^Institute for Global Food Security, School of Biological Sciences, Queen's University, Belfast, United Kingdom

**Keywords:** social behavior, welfare, contest behavior, social relationships, livestock

## Abstract

A fundamental understanding of behavior is essential to improving the welfare of billions of farm animals around the world. Despite living in an environment managed by humans, farm animals are still capable of making important behavioral decisions that influence welfare. In this review, we focus on social interactions as perhaps the most dynamic and challenging aspects of the lives of farm animals. Social stress is a leading welfare concern in livestock, and substantial variation in social behavior is seen at the individual and group level. Here, we consider how a fundamental understanding of social behavior can be used to: (i) understand agonistic and affiliative interactions in farm animals; (ii) identify how artificial environments influence social behavior and impact welfare; and (iii) provide insights into the mechanisms and development of social behavior. We conclude by highlighting opportunities to build on previous work and suggest potential fundamental hypotheses of applied relevance. Key areas for further research could include identifying the welfare benefits of socio–positive interactions, the potential impacts of disrupting important social bonds, and the role of skill in allowing farm animals to navigate competitive and positive social interactions. Such studies should provide insights to improve the welfare of farm animals, while also being applicable to other contexts, such as zoos and laboratories.

## Introduction

A fundamental understanding of behavior is essential for improving the welfare of billions of farm animals globally. Although artificial environments release animals from natural selection pressures, fundamental ethological principles remain relevant as farm animals carry an “evolutionary legacy” that influences behavior [(1); see also (2–5)]. For example, comparative studies have found that the behavioral repertoire of many farmed species has been qualitatively preserved since domestication [(6); e.g., chickens: (3); pigs: (7, 8)]. On the other hand, domestication has also quantitatively modified behavior in many farmed species by altering response thresholds (2, 9). These behavioral changes may be brought about intentionally through artificial selection, or inadvertently due to correlations between behavior and production-relevant traits (9). Although the process of domestication involves adaptation to captivity and management by humans, some behaviors that once enhanced fitness under natural conditions may compromise welfare in farm environments. For example, while a life in captivity releases animals from the need to find food and avoid predators, it may create additional challenges by housing animals at high population densities or in groups with an unnatural social structure (10). As a result, the behavioral strategies that allowed animals to navigate their social environment under natural conditions may be constrained, or no longer beneficial, in captivity (2, 10). Understanding the evolutionary origins and ontogeny of behavior, and how behavior is altered or constrained by the farm environment, is therefore central to improving welfare (1, 4, 5).

Although farm animals may not be able to control or modify their environment, they are still capable of making important behavioral decisions that influence their welfare (2, 10). Decision making may require overcoming the challenges of recognizing social companions, remembering previous interactions and attending to social cues (11). While farm animals have been shown to discriminate between conspecifics (12), how they cope with other challenges, such as recognizing their position in a dominance hierarchy or estimating the fighting ability (resource holding potential, RHP) of competitors, is not well understood. Moreover, farm social environments may exert additional challenges beyond those experienced by animals' wild ancestors [e.g., by housing animals in groups of the same sex, age and RHP, or in such large groups that individual discrimination is impossible; e.g., chickens (13), pigs (14)]. This impacts welfare as animals may compete for longer to establish new dominance relationships, and subordinate individuals are offered limited opportunities to escape. Indeed, acute and chronic social stress of farmed animals is a leading welfare concern (15, 16). Mitigating social stress therefore requires an understanding of how the farm social environment differs from the ancestral state, the social challenges associated with artificial environments, and how these challenges are overcome (1, 2, 12).

Farm animal systems have also been used to address fundamental questions regarding behavioral control and development, offering opportunities for longitudinal studies at large sample sizes with replication, and with a high degree of environmental control that may be difficult to achieve with wild animals. Studies of farm animals have contributed to our fundamental understanding of various aspects of animal behavior, including parent–offspring conflict (4, 7, 8, 17), signaling (18–20), foraging decisions (3, 21–25), and social learning (26–28). These studies illustrate how applying fundamental ethological principles to farm animals provides insights into the mechanisms underlying behavior, with findings being of relevance to wild animals but often having implications for the management and welfare of captive animals too (4, 7, 29). For example, both fundamental and applied ethologists share the common goal of understanding how and why individuals vary in their social behavior. Factors including age, sex, personality, cognitive ability, affective state, and previous experience may interact to generate inter-individual variation, influencing wild and farm animal behavior and the efficacy of welfare interventions (30–32). In particular, social skills [e.g., appropriately giving and interpreting social signals, performing behavioral responses appropriate to a given situation, responding to the behavior of conspecifics; (33, 34)] may play an important role in mediating social interactions in livestock. These skills may contribute to social competence by allowing individuals to optimize their social behavior across contexts (35). The development of social competence and skill is a key topic of interest in animal behavior research, from both applied and fundamental perspectives (34, 35). Studies of livestock not only lead to welfare benefits but also play an important part in improving our understanding of the control, development, and function of social skills by providing opportunities for large scale, carefully controlled experiments grounded in fundamental behavioral theory.

In this review, we highlight opportunities to build on existing work, where further integration of fundamental behavioral theory may benefit the study of farm animal welfare. As social interactions represent perhaps the most dynamic and challenging aspects of the lives of farm animals, we focus on agonistic and socio–positive interactions, and suggest potential fundamental hypotheses of applied relevance. While we focus primarily on farm animals, enhancing our fundamental understanding of social behavior may also provide insights to improve the welfare of managed animals in other captive settings, such as zoos and laboratories (36, 37).

## Agonistic interactions

Agonistic interactions serve an important function by influencing access to fitness-enhancing resources. In many animal societies, the outcome of these interactions results in relatively stable dominance relationships that reduce the need for frequent and costly aggressive interactions (38). Due to their ancestral socio–ecology, agonistic behavior and dominance relationships remain major features of the social lives of farm animals, even when the need to compete for resources is reduced. For example, domestic pigs, and to a lesser extent other livestock species, retain the motivation to establish dominance and will do so both in the absence of resources to compete over, or in the presence of adequate resources for all (39–41). However, in the context of the artificial farm environment, welfare issues arise if aggression is severe and/or persists in the long term. Aggression is a widespread problem in pig farming, and is associated with increased stress (42, 43) and injury risk (44), and reduced immune function (45). In turn, these welfare impacts have consequences for farm profitability and sustainability by compromising growth rates (46) and feed use efficiency (46, 47). Managing aggression in pigs is therefore of considerable research interest, with a wealth of empirical studies quantifying variation in aggressive behavior within and between groups [e.g., (41, 44, 48)]. Most experimental work to date has focused on the role of housing conditions and group size on aggression [e.g., (15, 49, 50)], and comparatively little is known about the fundamental drivers of the observed variation in aggressive behavior (2). Our research group has used predictions from game theory to better understand contest behavior and decision making in pigs, with the goal of improving welfare and providing fundamental insights into the mechanisms and development of contest behavior more widely.

### A game theoretical approach to studying competitive interactions

Insights from behavioral ecology may enhance our understanding of aggressive behavior in farm animals. In particular, game theory models have been widely applied to explain the evolution of contest behavior in wild species. Early game theory models sought to explain the evolution of contest behavior by comparing the relative fitness payoffs of aggressive and non-aggressive behavioral strategies, and how these strategies influence contest outcomes (51). Initial models set the scene for more realistic game theory models that considered asymmetries between contestants and the costs and benefits of obtaining information during contests (52). During a contest, individuals may gather information on the value of the resource being disputed (53), and their likelihood of winning based on factors such as size, skill, or weaponry [RHP; (52, 54)]. Contestants may also gather information on the RHP of their opponent (52). While gathering this information is likely to reduce uncertainty and allow individuals to only engage in contests that they are likely to win, it also carries costs (55). Selection may therefore favor the adoption of assessment strategies that dictate the information sources that individuals use in deciding when to escalate a contest, and when to withdraw (52). Several candidate models have been proposed, including: (i) resource-only assessment strategies, where decisions to withdraw are based on the value that each rival places on the contested resource (53); (ii) self-assessment strategies, where individuals rely on information about their own RHP and losers withdraw once their individual cost-bearing threshold is exceeded [e.g., war-of-attrition models, (56, 57); cumulative assessment model, (58)]; (iii) opponent-only assessment strategies, where individuals base their decision to withdraw on opponent RHP independently of their own RHP or fighting ability (52); and (iv) mutual assessment strategies, where individuals gather information regarding their own RHP relative to their opponent, and losers withdraw once they establish that they are the weaker rival (59). To distinguish between these potential models, empirical studies analyze how the costs of competitive interactions differ according to RHP of the defeated and victorious opponent (52). A recent meta-analysis determined that self-assessment strategies appear to be more common overall (60), while some studies provide robust evidence for mutual assessment [e.g., dragonflies *Diastatops obscura* (61); cuttlefish *Sepia apama* (62)]. However, most studies find no strong support for any single assessment strategy [e.g., the freshwater crustacean *Aegla longirostri* (63)], suggesting that they may not be mutually exclusive and instead represent variation along a continuum (64, 65). While the optimal assessment strategy is likely to differ according to ecology and life history (60), assessment strategies may also vary within species if different information is available to different opponents (52, 65). Furthermore, individuals may alter their assessment strategy over their lifetime, or even over a single contest (65–69).

Several factors may explain individual variation in contest behavior and assessment strategies. In some species, males and females differ in their contest behavior [convict cichlid *Amatitlania nigrofasciata* (70); jumping spider *Phidippus clarus* (71); brown anole *Anolis sagrei* (72)], and while the underlying mechanisms are not always clear, differences in life history may influence the cost of defeat and individuals' assessment of RHP and resource value [see (71)]. Motivation to engage in fights may also vary as individuals age: for example, older individuals may increase investment in contests that maximize their current reproductive opportunities, while younger individuals may prioritize the future survival over the current reproductive attempt [(73); but see (74)]. Age-related effects may also influence RHP through changes in body size, condition, or morphology (75), and over time, contest experience may allow individuals to develop fighting skills (33, 34). The outcomes of prior contests can generate winner and loser effects, whereby victories increase the probability of winning in subsequent contests, and defeat increases the probability of losing in future without directly affecting fighting ability (74, 76–78). Furthermore, consistent inter-individual variation in behavior, comprising traits such as boldness and aggressiveness, are likely to have an important influence on contest behavior and outcomes (79). Other factors, including affective state and cognitive ability, are also likely to contribute to individual variation in decision making during contests (80, 81). Affective state comprises short-term emotions and longer-term moods that influence responses to potentially rewarding or punishing stimuli (82). Specifically, an animal's affective state determines how stimuli in the environment are appraised based on prior experience, and is therefore likely to play a central role in contest behavior and decision making (81). Though studied in applied ethology as an indicator of welfare, the role of affective state has received little attention from behavioral ecologists and is yet to be formally integrated into contest theory (81). Likewise, the role of cognition in influencing contest dynamics and the development of fighting skills is an important avenue for future research (80, 83). Cognitive processes including perception, categorization, learning, and memory are vital to assessment and decision making and may form an important component of RHP (80, 83, 84). Several factors including diet, environmental enrichment, developmental stress, and personality may affect individual cognitive performance, and therefore decision making during contests (80, 85, 86). The effect of the social environment on cognitive development is a key area of interest in livestock studies (87–91), and these systems provide opportunities to design experiments investigating how social experience shapes assessment strategies and information use during contests (80).

### Applying game theory to improve farm animal welfare

To illustrate how a game theoretic approach can be applied to examining agonistic interactions in farm animals, we draw on previous work investigating contest behavior and decision making in pigs. Substantial variation in aggression persists at an individual and group level, associated with the establishment of dominance relationships between unfamiliar pigs and in competition for resources between familiar individuals (41, 44, 48). Identifying the mechanisms underlying this variation is the focus of considerable research effort. Aggression in pigs has a heritable genetic component [e.g., (92–95)], but is also influenced by social experience (40, 96, 97). Along with others (98), recent work from our group has used a game theoretical approach to explain variation in contest behavior in pigs, with a view to minimizing the costs of agonistic encounters.

Enabling litters to mix prior to weaning (termed socialization) allows pigs to resolve later life dominance disputes more quickly and at a lower cost, by increasing investment in display behavior rather than escalated fighting (40, 96). Allowing piglets to interact with a greater diversity of social companions from a young age may facilitate the development of later life social skills and assessment abilities, through activities such as play fighting (69, 99). Interestingly, the findings of these studies suggest that the effects of play fighting experience on contest outcomes differed between males and females, possibly due to sex differences in sociality and the costs of contests in the wild. While male piglets demonstrated more play fighting behavior than females (69, 100), play fighting experience only increased the probability of winning in contests between females, suggesting that play fighting may not prepare males for adult conflict (97). Early life socialization and play fighting experience may also determine the assessment strategy that pigs adopt during contests. Specifically, when pigs with socialization experience later engaged in a dyadic contest with an unfamiliar opponent, losers of lower RHP engaged in longer and more costly contests against stronger opponents (40, 97). This effect is consistent with a form of mutual assessment, albeit in the opposite direction to the initial predictions of mutual assessment models [which predict a negative relationship between winner RHP and fight duration (40)]. Despite being counter-intuitive, in some cases it may be beneficial for small individuals to engage in contests with larger opponents. For example, smaller individuals may be motivated to engage in fights if RHP is not the sole predictor of contest outcome [a “Napoleon strategy” (101, 102)], or if low-RHP individuals have few alternative options by which to gain resources [a desperado effect; (103)]. In this case, it implies that early life social experience allows pigs to develop higher confidence in their own RHP (97), possibly through improving assessment abilities (40). The effect of early life experiences on assessment strategies was further supported when subjects were matched against an opponent of similar play-fighting experience; pigs in dyads with more play-fighting experience appear to employ a self-assessment strategy, while those in dyads with less play-fighting experience showed no clear assessment strategy (104). Early life social experience also appears to influence the development of aggression as socialized piglets, particularly females, were quicker to attack a smaller opponent (69). Taken together, these findings suggest that experience of play fighting allows female pigs to signal their motivation to engage in a contest and thereafter resolve disputes quickly and with fewer costs. In contrast, it may be that males are less inclined to initiate aggression due to the higher costs associated with male-male contests in the wild (69). It is noteworthy that, in the specific and ecologically relevant context of a dyadic contest (as compared to reformation of the entire social group which is rarely seen in the wild), immature male domestic pigs show aggression that is more prolonged and 3.7 times more damaging than between females (68). Consequently, the legacy of male–male conflict in the wild may still be relevant to male domestic pigs and lead to their reluctance to rush into escalated fights. At least, this appears to be the case in these studies of entire male pigs: In many countries, pigs are still routinely castrated and while this appears to reduce overall aggression (105, 106), the most in-depth studies of contest dynamics in male pigs to date have focused on uncastrated males. Overall, these studies suggest that the welfare of domestic pigs is likely to be improved by management systems that promote early life social experience and reduce contest costs, but primarily in females. In addition to play fighting, experience of prior contests is also important in determining assessment strategy. We showed that naive pigs adopt a self-assessment strategy in their first contest, and a mutual assessment strategy in subsequent contests (68). However, after intensive fighting experience, individuals revert to a self-assessment strategy. This suggests that while previous fighting experience is useful in developing mutual assessment, individuals rely on estimates of their own RHP if this information is sufficiently reliable or the costs of obtaining information about the opponent are too high (68).

Game theory predicts the emergence of variation in aggressiveness which may be predictable over time and form a component of personality (79). In agonistic contests, aggressiveness may contribute to RHP and provide information to be utilized during assessment (79, 107). In line with other studies (108, 109), Camerlink et al. (107) found that aggressiveness in pigs did not predict contest outcome or the probability that a contest would escalate to a fight, but more aggressive opponents were likely to initiate the first attack. Consequently, aggressive individuals were more likely to win contests that did not escalate to a fight (107), suggesting that aggression may act as an honest “signal of intent” rather than a component of RHP. Aggressiveness also affects other aspects of contest behavior in pigs (110). Among the winners of contests, less aggressive winners spent more time in the pre-escalation phase of a contest compared to more aggressive winners, and showed fewer agonistic behaviors following their opponent's retreat. Failure to invest in pre-escalation assessment likely leads to unnecessary escalation whilst excessive aggression toward a defeated opponent likely contributes to the welfare issues associated with contests. However, in subsequent contests, winner–loser effects appeared to have a stronger influence than aggressiveness on the initiation of agonistic behavior, suggesting that both more- and less-aggressive pigs integrate past experience during contest decision making (78). Taken together, these findings suggest that management or breeding approaches which reduce aggressiveness would be expected to benefit welfare without preventing the effective establishment of dominance relationships, or the ability of individuals to learn from previous contest experience.

Other personality traits, such as boldness and impulsivity, along with factors such as cognitive ability and affective state, may be important in farm animal contests (79–81). For example, bold individuals may be more willing to take risks that help them to win fights (79). Similarly, more impulsive individuals may be more likely to initiate fights, potentially influencing contest outcome (84). Aspects of personality, cognition, and affective state are widely studied in livestock species, and have been shown to vary between the individuals (85, 111–113). Currently, the importance of personality, cognition and affective state in determining information use and RHP during agonistic interactions is not well understood [but see (84) and (108)]. Future work in this area has the potential to create new understanding of the fundamental determinants of contest behavior and improve welfare in pigs by identifying how individual characteristics influence assessment strategies and dominance. This knowledge could be applied to minimize the cost of agonistic interactions, by informing optimal group composition or facilitating the development of social skills. To date, the majority of game theoretic studies in farm systems have focused on pigs and fowl [e.g., (114)], with all research on contest assessment strategies in domesticated species focusing on pigs. Although aggression in other livestock species is less severe, studies of a broader range of species may provide insights into the generality of the proximate mechanisms underlying agonistic behavior, and their welfare implications for animals kept in captivity.

## Positive social interactions

In addition to competitive interactions, positive interactions also form part of an animal's “social world.” Positive interactions with conspecifics have been shown to be beneficial in a range of social species (11, 115). For example, studies of non-human primates (116–118), cetaceans (119–121), and horses (122) demonstrate that individuals with more and/or stronger affiliative bonds benefit from enhanced survival or reproductive success in the wild. Precisely how social bonds increase fitness varies depending on the socio–ecology of the species in question. While social bonds are often formed between close kin, bonds between non-relatives also occur in some animal societies (123) and may be associated with fitness benefits [e.g., feral horses (122); vampire bats (124); bottlenose dolphins (125); greater ani (126); Assamese macaques (127); house mice (128)]. Farm animals may also be motivated to form and maintain relationships with specific social companions, if these relationships are beneficial (30). For example, some farmed species may form social relationships if these served an important function in their pre-domesticated evolutionary history, and/or because they continue to derive benefits from these interactions in a captive setting. Positive social interactions have the potential to enhance welfare, but have received comparatively less research attention than negative social interactions, such as aggression (30, 129).

While many farm animals appear to benefit from positive interactions with conspecifics [see (30) for a review], whether and how individuals benefit from interactions with specific social companions is less well studied. The mother–offspring bond often has an important influence on offspring health and welfare in a wide range of species, particularly in mammals (11, 115), and understandably, mother–offspring bonds have been a strong focus for farm animal research. Studies in cattle, for example, have shown that cows are highly motivated to return to their calf after a period of separation (130, 131), and that access to the dam has positive effects on calf social development [(132), see also (133, 134)]. This suggests that maternal contact in farm animals informs the development of social behavior, and raises the possibility that the early weaning age associated with many livestock systems may constrain positive behavioral development. Access to social companions, particularly the dam, influences the response of young farm animals to stressful or challenging events [e.g., calves (135–137); goats (138)]; and in cattle, familiar social companions provide more effective social support than unfamiliar conspecifics (139, 140). However, the importance of individualized social relationships beyond the mother-offspring bond has received comparatively less research attention. Within social groups, preferential associations have been observed in cattle (141, 142) and goats (143) and to a lesser extent in pigs (144–146) and sheep (147). Some species also engage in behaviors such as allogrooming [e.g., cattle (142)] and social nosing [e.g., pigs (148)]. The exact function of these behaviors in farm animals is not fully understood, but current evidence suggests that they may provide various benefits to actors and/or recipients [reviewed in (30, 129, 149)]. For example, it has been suggested that social grooming may play a role in maintaining dominance relationships and social cohesion in cattle (150), and reduce social tension in competitive situations (142). While allogrooming and similar behaviors in farm animals are often interpreted as affiliative, the extent to which they represent a reciprocal or preferential social relationship is unclear (142). Further work investigating whether farm animals form socio–positive relationships with particular social companions, how these relationships are formed and maintained, and how they influence welfare, would be very valuable (30). It may be that the apparent welfare benefits that farm animals derive from socio–positive interactions are a legacy of the drivers that enhanced fitness in these species' pre-domesticated evolutionary past. To this end, studying the functions of socio–positive interactions in closely-related wild species may yield valuable insights as to why livestock may be motivated to maintain these relationships in captivity.

Few empirical studies have investigated how social relationships are initially formed in non-human animals (11). Not all individuals form preferential relationships to the same extent, and likewise, positive social interactions may benefit individuals in different ways. These differences can arise for several reasons. Firstly, animals may prefer to associate with familiar individuals, as seen in cattle and pigs (151, 152). Kin-based associations (e.g., between sisters) have also been identified in closely-related members of wild species [e.g., collared peccary *Pecari tajacu* (153); wild boar *Sus scrofa* (154); European bison *Bison bonasus* (155)], and many farm animals form preferential relationships with related and unrelated individuals [reviewed in (129)]. In this way, the “unnatural” regrouping of unfamiliar animals on farms may result in separation of preferred social companions, creating welfare problems in addition to those associated with regrouping aggression. Sex differences may also play an important role; in many species, the philopatric sex invests in social relationships to a greater extent, as these relationships may be of greater fitness relevance [e.g., (156–159)]. Few studies have investigated whether male and female farm animals differ in their tendency to form socio-positive relationships, and how these differences may be explained in terms of their ancestral ecology. Further research in this area would help to identify and explain individual variation in behavior in captivity. The benefits and nature of social bonding may also vary with age. Longitudinal studies in rhesus macaques (*Macaca mulatta*), for example, have shown that social relationships appear to be closely tied to survival, particularly for younger individuals [(160); see also (161)]. How and why individuals' social relationships vary with age is not fully understood but may provide insights that aid our understanding of the social structure of farm animals. Few studies have investigated age effects on social associations in farm animals, although reproductive status appears to influence associations in female cattle and sheep [possibly driven by offspring behavior (162, 163)]. In this case too, an understanding of the function and evolutionary history of socio–positive relationships in ancestral species would be useful to determine the importance of these interactions in livestock across the life course.

Finally, individual differences in social behavior, underpinned by variation in personality, previous experience and decision making, may affect the formation of social relationships. The role of personality in relationship formation has been studied in other species [e.g., (164–167)], providing some evidence that individuals may form preferential associations or relationships based on homophily in personality. How personality specifically influences social relationship formation has yet to be tested in farm animals, although some studies find links between personality measures and general sociality or social motivation [e.g., (168)]. In the field of animal behavior more widely, there is growing interest in identifying the behavioral skills involved in navigating socio–positive interactions (34), the factors underpinning variation in these skills [e.g., cognition (32, 34)], and how the ability to form socio–positive relationships correlates with performance in other social contexts (such as agonistic interactions) to contribute to social competence (34, 35). As a result, studies investigating how farm animals vary in their tendency to form positive social relationships may provide valuable insights into the mechanisms and development of behavior in addition to the potential implications for enhancing welfare.

Identifying the benefits of positive social interactions has attracted growing interest from behavioral ecologists in recent years, and developments in this field may be useful to enhance our understanding of social relationships in farm animals. Studies in wild animals have identified some of the functional benefits of social relationships (116–122), how these benefits vary according to species' ecology (118, 120–122) and the proximate mechanisms underlying social structure (161, 165, 169). While the majority of studies to date provide correlative rather than causative evidence, some studies have begun to test hypotheses relating to social structure through experimental manipulations of social groups (170). For example, studies in Paridae have used RFID technology to manipulate which members of a population gain access to foraging opportunities. These experiments showed that social relationships have wide-ranging effects on individual behavior and decision making. First, the social associations formed during foraging appear to carry over to other situations, demonstrating that the effects of social network disruption may extend beyond the immediate behavioral context in which the disruption occurs (171). Furthermore, individuals were willing to forego feeding opportunities to maintain important social relationships (172), and individuals responded to the loss of a close social associate by increasing their connections to the wider network (173). Automated monitoring systems are increasingly being used on farms to analyze space use, activity budgets and spatial associations, and are being developed to automatically distinguish between affiliative and agonistic social interactions [e.g., (151, 174–176)]. Similarly, automated systems have been used to control animals' access to resources in farm systems, in order to manipulate social interactions [e.g., in cattle (177, 178)]. Motivation tests are also widely used to assess the behavioral needs of farm animals (179), including motivation to obtain contact with particular social companions [e.g., (130, 180)]. There may be opportunities to make further use of these methods to conduct controlled experiments determining the extent to which individuals are willing to invest in social relationships with different individuals within their social group, and how these relationships influence behavior and welfare across contexts. By understanding the value of positive social relationships in farm animals, we can identify the welfare impacts of separating individuals from preferred social companions. For example, a recent preliminary study found that separating dairy cows from their preferred partner increases variability in milk yield, a possible indicator of stress (177). In some situations, the impacts of separating preferred partners could be severe; on the other hand, the loss of a social companion may be mitigated if individuals compensate by strengthening relationships with other group members. While controlled experiments could be used to address these questions, social disruptions also occur frequently during on-farm activities, and these perturbations provide opportunities to investigate the speed and ease with which new positive social relationships are formed and their influence on welfare and productivity [e.g., (177, 181)]. These studies could also be used to identify “keystone” individuals that have a disproportionate influence on group-level behavior. While this idea has drawn interest in the context of harmful social behavior in farmed animals (99, 182–185), it has yet to be tested in the context of affiliative behavior [but see (152)]. The farm system provides a highly controlled environment in which to investigate the dynamics of positive social interactions, the formation of preferential relationships, and the potential welfare implications.

## Future directions

In this section, we suggest key questions for future research and highlight potential hypotheses of both fundamental and welfare relevance. We summarize our ideas in Table 1, using three of Tinbergen's four questions (function, mechanism, and development) as a framework.

**Table 1:** Summary of potential avenues for future research.

**Tinbergen's questions**	**Research questions**	**Methods**	**Welfare-relevant hypotheses**	**Examples**
Ancestral function of social behavior, effects of domestication and relevance for farm animal welfare	• How do contest resolution skills improve welfare in farm environments? • How do socio-positive interactions improve welfare in farm environments? • Has domestication inhibited the development of social skills in farm animals?	• Behavioral observations • Controlled experiments • Social network analyses (individual and group-level metrics) • Longitudinal studies • Comparative studies of closely-related wild species	1. Contest resolution skills allow individuals to resolve conflict more efficiently with fewer costs 2. Separating preferred social partners compromises welfare	1. (40, 69, 97) 2. (135, 139, 140, 177, 186, 187)
Mechanisms underlying social behavior	• Which factors, separately and interactively, generate variation in social behavior between individuals? - Personality - Cognition - Affective state - Sex - Age - Reproductive status	• Behavioral observations • Social network analyses • Experimental manipulations of relevant characteristics	1. Cognitive skills facilitate contest decision-making and assessment 2. Affective state influences judgement and decision-making during contests 3. Motivation to engage in contests varies according to sex and age 4. Motivation to form social bonds varies with sex and age 5. Factors such as cognitive ability, personality and affective state influence the formation and development of social relationships	1. (84) 2. (84) 3. (69) 4. (162, 163)
	• How does individual variation in social behavior influence group-level transmission patterns?	• Social network analyses Network-based diffusion experiments	1. Social structure and group composition influence the spread of behavior through groups (affiliative behavior, agonistic behavior, and other welfare-relevant behaviors e.g. tail-biting in pigs)	1. (49, 99, 183–185, 188, 189)
	• How do farm animals communicate during social interactions?	• Signaling during agonistic and affiliative interactions • Experimental manipulation of contest signals • Communication during e.g. social preference tests • Effect on receiver behavior	1. Farm animals use signals of resource holding potential (RHP) and motivation during competitive interactions 2. Farm animals direct communication toward preferred social partners	1. (107) 2. (18, 190–192)
Development of social behavior	• How do social skills contribute to RHP and assessment strategy? • How do individuals vary in their contest resolution skills? • How does prior experience influence acquisition of contest skills?	• Staged dyadic contests • Systematically vary RHP between opponents • Quantify individual differences in skill • Manipulate early life social and/or fighting experience	1. Social skills allow farm animals to resolve conflict more efficiently 2. Early life experiences can be manipulated to encourage development of relevant social skills and improve welfare 3. Maternal deprivation impairs development of social skills and compromises welfare in later life e.g. costly contest behavior	1. (40, 69, 78, 97, 104, 193) 2. (40, 69, 97, 193)
	• Which skills/behaviors are involved when new relationships are formed? • How does previous experience facilitate acquisition of social skills? • How do individual relationships change over time?	• Quantify relationship strength e.g. social preference tests • Choice of social companions • Manipulate social associations and relationship formation	1. Social skills facilitate formation of socio-positive relationships that enhance welfare 2. Maternal care facilitates development of social skills 3. Social skills influence the ability of farm animals to adapt to changes in their social environment	
				2. (132–134) 3. (99, 135, 136, 186, 194, 195)

### Ancestral function of social behavior, effects of domestication and relevance for farm animal welfare

Knowledge of the ancestral functions and origins of behavior can contribute to our understanding of farm animal social behavior, including how a history of artificial selection has altered behavioral expression, and how behaviors that may have historically enhanced fitness in the wild translate to the captive farm setting. For example, has the expression of contest skills in farm animals been altered by artificial selection and/or the nature of the farm environment? What relevance might these changes have for animal welfare? While the consequences of farm animal agonistic interactions are relatively well documented overall, there are many opportunities to identify important skills and the mechanisms influencing their development. Moreover, comparatively fewer studies have investigated the benefits of socio-positive interactions for farm animal health and welfare (30, 129). Social preference tests and experiments that measure individuals' motivation to return to specific group members may be particularly useful in addressing these questions [e.g., see (130, 140, 180); see Table 1]. Investigating the emotional costs of breaking social bonds may also provide valuable insights into the function and benefits of positive social interactions. For instance, judgement bias tests are widely used in applied ethology to quantify changes in affective state (112, 196), and may be employed to investigate the emotional responses of animals separated from preferred social companions. As most studies of socio-positive interactions in farm animals focus on short-term benefits, it would be especially valuable to extend studies to assess long-term effects on animal health and wellbeing and the costs of separating socially bonded individuals (30).

### Proximate mechanisms underlying behavior

How individual variation in cognition, affective state, and personality interact to influence social skills and decision making in farm animals forms a key area for future research, with potential implications beyond managed species. Examining individual differences in social behavior, and the drivers of this variation, may be useful in influencing how group composition determines patterns of aggression and affiliation, and how behaviors are transmitted through groups. Technological developments have resulted in social network experiments becoming increasingly popular in the fields of both behavioral ecology and applied ethology, often incorporating manipulations of group characteristics to provide a wealth of information about social dynamics. Social network studies are being applied to understand patterns of affiliation, aggression and the spread of problematic behaviors in farm animals (197, 198), and there are opportunities to build on previous studies by manipulating interactions within social networks, either as part of controlled experiments or existing management activities.

There are also opportunities to learn more about the communicative mechanisms by which farm animals mediate their social interactions (1). While there is a body of work focusing on mother-offspring communication in farm animals [e.g., (18, 19, 190–192)], less is known about communication and signaling between peers, particularly during socio-positive interactions. Do individuals communicate with all group members, or direct signals toward preferred social companions? In the latter case, communication patterns may provide important information about the nature and strength of social relationships. For example, pigs vocalize when separated from their peer group [e.g., (199)]; while this appears to be a clear indicator of social isolation stress, it would also be useful to determine whether these vocalizations are more frequently targeted toward particular peer-group members, and how they influence receiver behavior. Farm animals may also communicate during agonistic interactions, providing signals of RHP, motivation or skill that facilitate decision making. Currently, very little is known about signals of RHP in farm animals. More work is needed to identify the relevant signals mediating these interactions, and how individuals interpret these signals. Beyond their role in mediating social interactions, vocalizations and other signals are often important indicators for welfare (1, 29, 129, 200, 201). Important insights can therefore be gained from identifying the functions of different signals and how the frequency of signaling varies according to management conditions (29).

### Development of behavior

The development of social skills is attracting growing interest in animal behavior research (33, 34), and empirical studies testing theories on the development of skill in farm animals would be both valuable and timely. Evidence that early social experiences shape contest behavior in pigs provides a useful model to test the role of skill and its development, and further research in other species would help to establish how skill influences contest outcome and assessment strategy. Following the predictions of game theory, these studies could identify how skills (including choice of behavior, accuracy, precision and efficiency) contribute to RHP, and quantify inter-individual variation in skill acquisition based on prior experience (33). It would also be valuable to further examine the role of social stability in influencing contest dynamics. In the case of pigs, while some studies show that maintaining stable social groups reduces aggression and benefits welfare (202, 203), long-term social stability is not common in most farm production systems. Interestingly, it may be that some degree of aggression is necessary for pigs to establish dominance relationships in unfamiliar groups, and an initial period of acute aggression after regrouping may lead to social stability being achieved more quickly (204). Identifying and encouraging the development of social skills may allow pigs to navigate these periods of acute aggression more successfully and lead to the establishment of stable dominance relationships with fewer costs. Further work could investigate the dynamics of aggression in livestock groups comprised of skillful individuals, and how these dynamics compare to less skillful groups, and groups that remain stable over a long period of time.

In addition to influencing agonistic interactions, social skills may play an important role in farm animals' affiliative interactions. Which skills are involved in forming beneficial social relationships? How do early life experiences facilitate the acquisition of these skills? Is there a key developmental window for the acquisition of particular social skills in farm animals (96, 205)? These questions could be addressed by identifying the specific behaviors involved in forming and maintaining social relationships in different species, and how individual social relationships change over time. More information is also needed on how farm animals choose their preferred social companions, based on factors such as familiarity, age, and personality. Longitudinal studies would be particularly valuable in this regard, allowing researchers to manipulate social associations, observe the formation of new social relationships and identify the relevant behaviors involved in mediating positive social interactions. By identifying the relevant skills farm animals use to navigate their social world, we can begin to investigate how individuals vary in their ability to choose the most appropriate behavior in different social contexts, and how this contributes to social competence (33–35).

## Conclusions

In this review, we have outlined how a deeper fundamental understanding of social behavior in farm animals can inform management and welfare. Important areas for further research include identifying the behavioral skills farm animals use to navigate competitive and positive social interactions; the factors underlying variation in skill; how different skills allow individuals to maximize the benefits arising from social interactions; and how a lack of skill contributes to welfare problems resulting from harmful social interactions. Exploring the welfare benefits of socio-positive interactions, and the potential costs associated with the disruption of important social bonds, also presents an important avenue for future research. Further increasing collaboration between fundamental and applied ethologists may provide opportunities to build on existing research, to mitigate social stress in livestock and address relevant questions regarding the mechanisms and development of social behavior. Similarly, the findings of these studies may provide insights that could be used to improve the welfare of managed animals in other contexts.

## Author contributions

VL wrote the manuscript with input and guidance from GA and ST. All authors contributed to the article and approved the submitted version.

## Funding

This project was funded by a BBSRC grant awarded to ST and GA (SRUC: BB/T001046/1; QUB: BB/T000716/1). SRUC also receives support from the Scottish Government Strategic Research Programme.

## Conflict of interest

The authors declare that the research was conducted in the absence of any commercial or financial relationships that could be construed as a potential conflict of interest.

## Publisher's note

All claims expressed in this article are solely those of the authors and do not necessarily represent those of their affiliated organizations, or those of the publisher, the editors and the reviewers. Any product that may be evaluated in this article, or claim that may be made by its manufacturer, is not guaranteed or endorsed by the publisher.
